# Adenosine and adenosine receptors: a “double-edged sword” in cardiovascular system

**DOI:** 10.3389/fphar.2025.1538680

**Published:** 2025-07-03

**Authors:** Yongqi Qian, Yixuan Zheng, Liang Leng, Qingqing Liu, Xiaojuan Tian, Shilin Chen, Sanyin Zhang, Jiang Xie

**Affiliations:** ^1^ School of Basic Medical Sciences, Chengdu University of Traditional Chinese Medicine, Chengdu, China; ^2^ The Third People’s Hospital of Chengdu, Clinical College of Southwest Jiao Tong University, Chengdu, China; ^3^ Institute of Herbgenomics, Chengdu University of Traditional Chinese Medicine, Chengdu, China; ^4^ Innovative Institute of Chinese Medicine and Pharmacy, Chengdu University of Traditional Chinese Medicine, Chengdu, China

**Keywords:** adenosine, adenosine receptors, cardiovascular system, contradictory, target

## Abstract

Adenosine serves a variety of biological purposes in the circulatory system and was first discovered in the heart in 1929. By interacting with four adenosine receptor (AR) subtypes of G protein-coupled receptors—A_1_AR, A_2a_AR, A_2b_AR, and A_3_AR—adenosine controls physiological processes. In pathological situations, spikes in adenosine activate the four receptor subtypes and alter downstream pathways by altering the generation of cyclic adenosine monophosphate, which contributes to autophagy and inflammation. There will inevitably be conflicting reactions from the various subtypes in this situation. Additionally, via mediating distinct signals or under various models and pathophysiological situations, the same subtype itself may have contradictory effects. Taken together, ARs’ conflicting regulatory roles in the cardiovascular system not only highlight the intricacy of their physiological roles but also offer a crucial avenue for future study into the treatment of cardiovascular diseases. The contradictory regulatory roles of adenosine and ARs in cardiovascular disorders, as well as their potential as therapeutic targets, are methodically outlined in this review.

## 1 Introduction

Cardiovascular diseases (CVDs), which include ischemic heart disease, heart failure, peripheral artery disease, and various other cardiac and vascular conditions, are the leading cause of death globally (GBD 2017 DALYs and HALE Collaborators, 2018; GBD 2017 Causes of Death Collaborators, 2018). Adenosine is a derivative of adenosine triphosphate (ATP) that has a significant impact on the cardiovascular system. The discovery of adenosine as a signaling molecule occurred in 1929 when an adenine molecule that lowers heart rate was found in heart tissue extracts. This metabolite was thought to be adenosine by Drury and Szent-Györgyi (Drury and Szent-Györgyi, 1929). Under normal circumstances, adenosine can be made and transferred out of cells; under stress, inflammation, and tissue damage, it can also be formed by the catabolism of adenine nucleotides (Koupenova et al., 2012; Yang et al., 2008; St Hilaire et al., 2008). Released adenosine activates four subtypes of adenosine receptors: A_1_AR, A_2a_AR, A_2b_AR, and A_3_AR. Different genes that are differentiated based on their affinity for adenosine encode these four kinds. In the heart, A_1_AR is mainly linked to Gαi and has a strong affinity for adenosine. The binding of deuterated 8-cyclopentyl-1,3-dipropylxanthine (DPCPX) to bovine cardiac membranes was the first evidence of A_1_AR expression in the mammalian ventricular myocardium. This suggests that DPCPX is a selective antagonist for the A_1_AR (Lohse et al., 1987). The same technique was then used to demonstrate A_1_AR expression in isolated rat ventricular myocytes (Martens et al., 1988). Additionally, radioligand binding experiments have confirmed that both coronary artery endothelial cells and coronary artery smooth muscle cells contain the A_2a_AR receptor, which has a high affinity for adenosine (Olanrewaju and Mustafa, 2000). A_3_AR can be linked to Gαi and Gαq and has a poor affinity for adenosine. Although A_3_AR expression in rat hearts was initially documented in 1992, the heart’s degree of expression is modest. (Zhou et al., 1992). In mammalian heart tissue, A_2b_AR is the fourth subtype of AR. The expression of A_2b_AR in mouse ventricular myocytes was confirmed by RT-qPCR (Chandrasekera et al., 2010). But activating ARs can have both positive and negative effects, which emphasizes the need for a more complex understanding of how ARs function in CVDs.

## 2 Adenosine formation and metabolism

Intracellularly, adenylate cyclase (AC) transforms ATP into cyclic adenosine monophosphate (cAMP), which is subsequently changed into AMP by phosphodiesterase (PDE). Ecto-5′-nucleotidase (CD73) then enzymatically cleaves AMP to provide adenosine. The cAMP-adenosine route is the biological process by which cAMP is converted into necessary adenosine (Tresguerres et al., 2011). The second intracellular pathway generates adenosine through the hydrolysis of s-adenosyl homocysteine (SAH) by SAH hydrolase (Camici et al., 2018).

**FIGURE 1:**
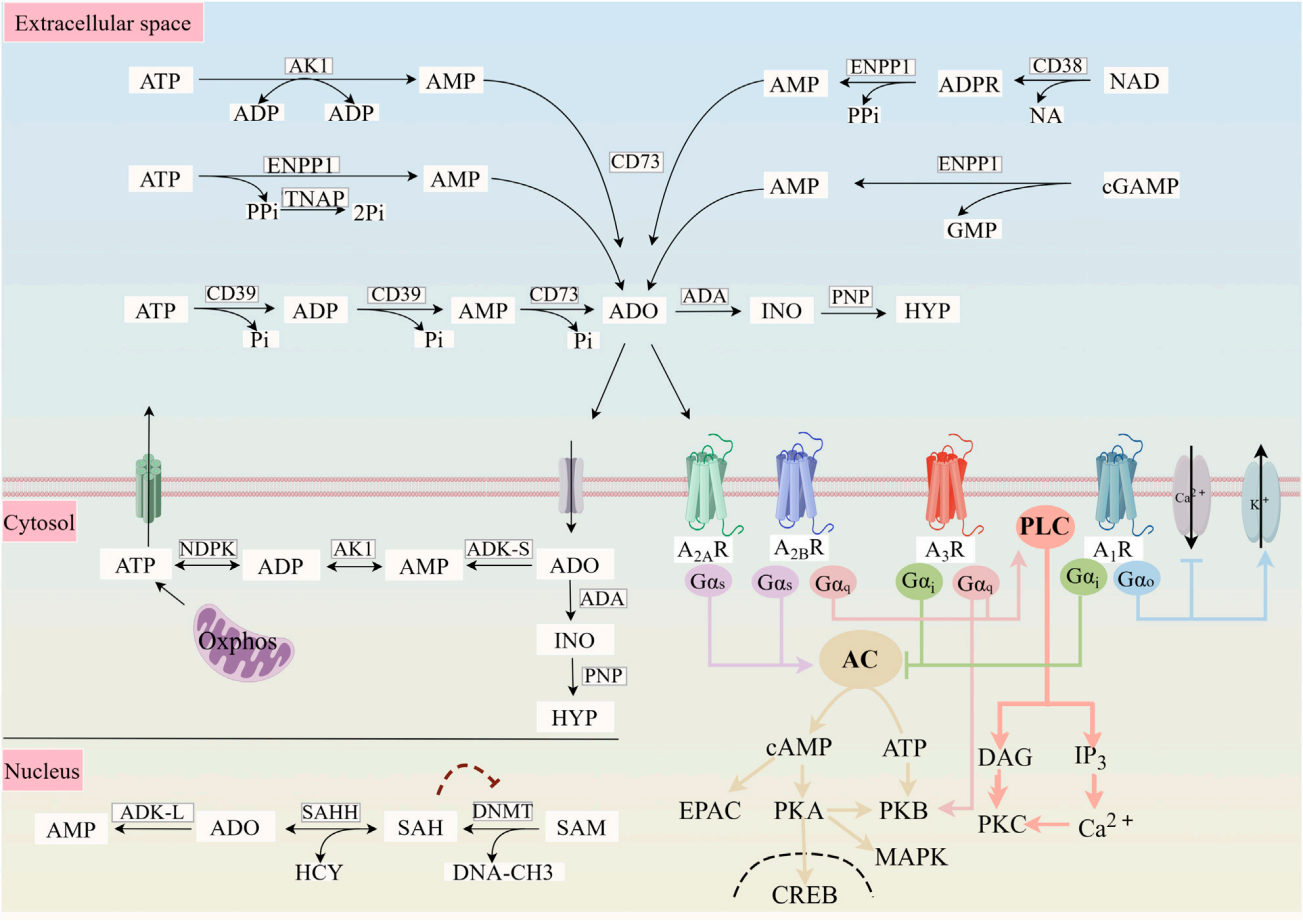
Adenosine production and metabolism and activation of ARs.

Extracellular triphosphate diphosphate hydrolases 1 (CD39) and CD73 sequentially dephosphorylate ATP to produce adenosine extracellularly, controlling adenosine availability. After being released extracellularly, CD39 dephosphorylates ATP from both phosphates to produce AMP, which is then dephosphorylated to become adenosine when extracellular CD73 is present. The generation of AMP can also be aided by other exoenzyme routes. For instance, ectonucleotide pyrophosphatase phosphodiesterase 1 (ENPP1) directly converts ATP to AMP and pyrophosphoric acid, while adenylate kinase 1 mediates the conversion of adenosine diphosphate to ATP and AMP (Dzeja and Terzic, 2009; Linden et al., 2019). Adenosine is produced from extracellular nicotinamide adenine dinucleotide and cyclic guanosine monophosphate adenosine monophosphate via two different pathways: the CD38-ENPP1-CD73 axis and the ENPP1-CD73 axis (Figure 1) (Gasparrini et al., 2021; Carozza et al., 2020).

Only momentarily present in the interstitial environment, extracellular adenosine is an intermediate metabolite of the nucleotide hydrolase chain. Through sodium-dependent concentration nucleoside transporters and sodium-independent equilibrium nucleoside transporters, cells quickly absorb extracellular adenosine for later metabolism (Allard et al., 2020). Adenosine can be quickly taken up by endothelial cells, erythrocytes, and surrounding tissues, pass the plasma membrane, and be used intracellularly. For instance, after being absorbed by endothelial cells, adenosine is either broken down by adenosine deaminase to inosine for use in the metabolism of uric acid or phosphorylated by adenylate kinase to generate AMP. The uptake of adenosine into the cell by these transporters marks the end of AR-mediated activity. Therefore, adenosine deaminase activity, the nucleoside transporter system, and intracellular and extracellular adenosine metabolism work together to terminate extracellular adenosine signaling.

## 3 Adenosine receptors in cardiovascular system

**FIGURE 2:**
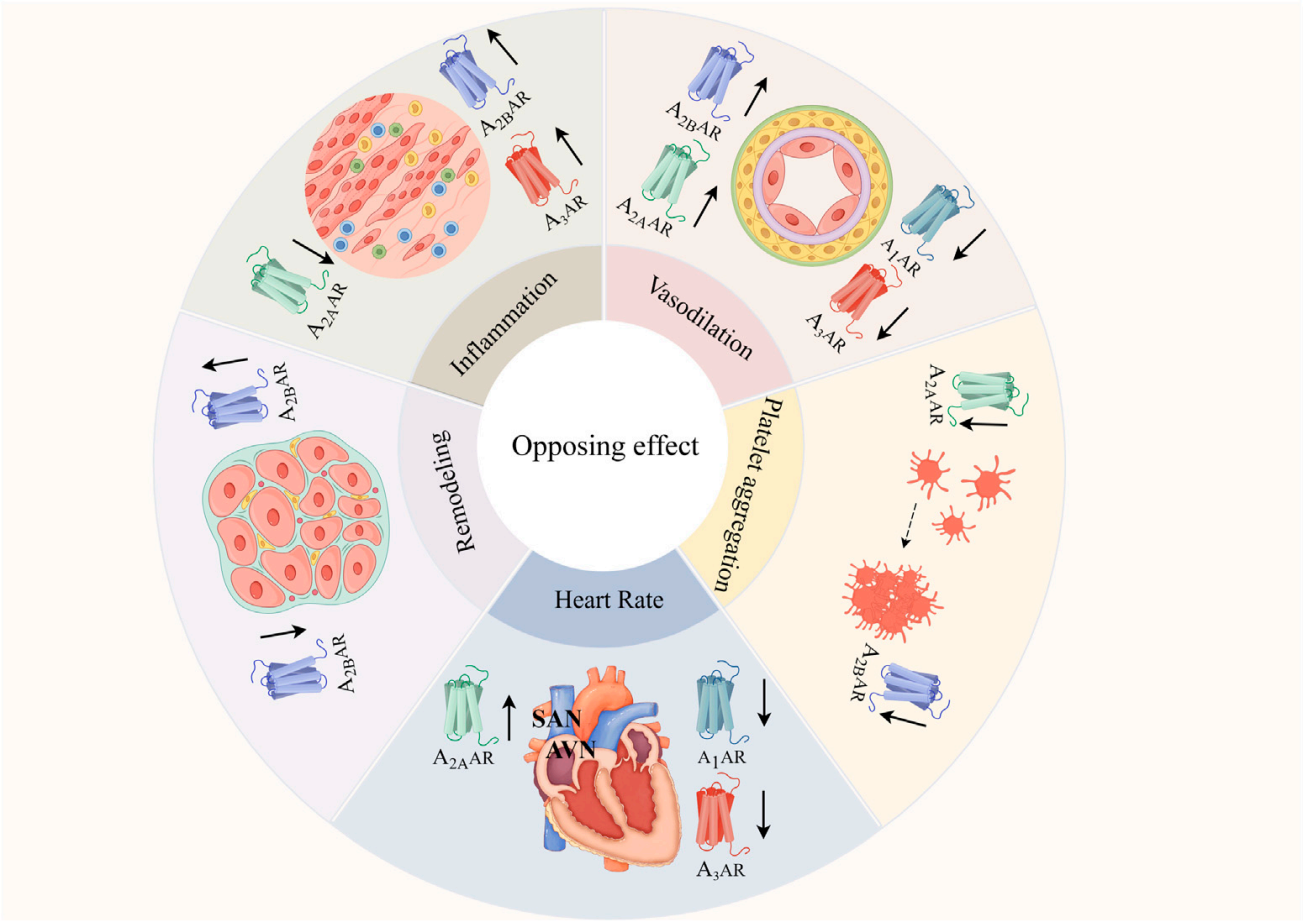
ARs’ contradictory functions in the cardiovascular system.

Adenosine, both inside and outside the cell, can be produced through a variety of pathways and activate ARs to function in the body. Signal transduction of ARs is largely G-protein-dependent and plays a broad and complex role in the cardiovascular system, and this complexity is mainly reflected in the opposing roles of receptors (Figure 2).

### 3.1 Signal transduction

ARs are members of the A-class of rhodopsin-like receptors, which bind to adenosine to produce their downstream actions. Based on the order of discovered and the distinct reactions upon activation, ARs are divided into four subtypes: A_1_AR, A_3_AR, A_2a_AR, and A_2b_AR, (Haskó et al., 2018; Sheth et al., 2014) (Table 1). A_1_AR and A_2a_AR are active in the nanomolar range, A_2b_AR and A_3_AR are active in micromolar concentrations, and A_2b_AR has the lowest affinity for adenosine (Sheth et al., 2014; Jacobson and Gao, 2006). AC catalyzes the conversion of ATP to cAMP. A_1_AR and A_3_AR couple to Gα_i_ to inhibit AC activation, while A_2a_AR and A_2b_AR coupled to Gα_s_ to activate AC (Zhou et al., 2020). A_2b_AR and A_3_AR also mediate the activation of phospholipase C through activation of Gα_q_, which increases diacylglycerol, inositol trisphosphate, and intracellular Ca^2+^. In addition, A_1_AR can be coupled to the pertussis toxin-sensitive Gα_o_ protein coupled with the activation of ion channels. Through their modulation of cAMP and Ca^2+^ levels, ARs contribution to the cardiovascular system. Protein kinase A (PKA), exchange protein, and cyclic nucleotide-gated ion channels are the three main targets that cAMP activates (Borea et al., 2018). The phosphatidyl signaling pathway is a double messenger system that generates inositol triphosphate and diacylglycerol, which trigger a cascade reaction within the cell by releasing and interacting with calcium ions (Borea et al., 2018).

**TABLE 1:** Classification and mechanism of ARs.

Receptor subtype	A_1_AR	A_3_AR	A_2a_AR	A_2b_AR
Chromosomal location	1q32.1	1p13.3	22q11.2	17p11.2–12
G protein coupling	Gα_i/o_	Gα_i_, Gα_q_	Gα_s_	Gα_s_, Gα_q_
Adenosine affinity	1–10 nM	0.1 μM	30 nM	1 μM
Effector	AC Ion channels: K^+^, Ca^2+^ Phosphoinositide 3-kinase Mitogen-activated protein kinase PKC	AC Phospholipase C Phosphoinositide 3-kinase Mitogen-activated protein kinase K_ATP_ PKC	AC Mitogen-activated protein kinase	AC Phospholipase C Mitogen-activated protein kinase
Effect on AC	Inhibitor	Inhibitor	Stimulation	Stimulation

The mitogen-activated protein kinase (MAPK) family, which includes extracellular signal-regulated kinases, p38, and Jun NH_2_ terminal kinases, can include the A_1_AR in its intracellular phosphorylation cascade response process (Schulte and Fredholm, 2000; Schulte and Fredholm, 2003). Glycogen synthase kinase 3 is increased when the A_3_AR is active because it lowers cAMP. β-catenin, cyclin D1, and c-myc will all be downregulated at the same time, and nuclear factor κB’s capacity to bind DNA will correspondingly decline. Additionally, the MAPK, nuclear factor-κB (NF-κB), and phosphatidylinositol-3-kinase-protein kinase B (PI3K-PKB/Akt) signaling pathways are all regulated by the A_3_AR (Borea et al., 2015). The most prevalent effector in the event of A_2a_AR activation is cAMP-dependent PKA, and the A_2a_AR also regulates the MAPK signal. Notably, the A_2a_AR may also be dependent on its lengthy COOH terminus. It interacts with auxiliary proteins such as α-actin, ubiquitin-specific protease, d_2_-dopamine receptor, ADP-ribosylation factor nucleotide site opener, and Translin-related protein X (Baraldi et al., 2008; Chen et al., 2013). Likewise, the A_2b_AR receptor has the ability to phosphorylate PKA and trigger the phosphorylation of c-Jun n-terminal kinase 1/2, extracellular signal-regulated kinase 1/2, and mitogen-activated protein kinase p38 (Merighi et al., 2017).

### 3.2 Function

#### 3.2.1 A_1_ adenosine receptor

The A_1_AR is mainly distributed in the sinoatrial node, atrial myocardium, atrioventricular node, and Hippocratic Purkinje system (Musser et al., 1993). Activated A_1_AR induced direct negative chronotropic, dromotropic, and indirect antiadrenergic effects, which counteracted the positive inotropic effects of catecholamines. A1AR causes a slower heart rate by suppressing the sinoatrial node and slowing down atrioventricular conduction. For patients with stable hemodynamics, intravenous adenosine is the drug of choice in the clinical treatment of arrhythmia (Katritsis et al., 2017). Ischemic preconditioning (IPC) is also associated with A_1_AR. Adenosine is one of the three autocrine signaling molecules released by ischemic tissues and is an important trigger of IPC. Activation of A_1_AR during hypoxia attenuates myocardial injury. Overexpression and deletion of A_1_AR led to a corresponding increase and decrease in myocardial resistance to ischemia (Matherne et al., 1997; Reichelt et al., 2005). Pharmacological preconditioning of male and female hearts with the A_1_AR agonist n6-cyclohexyl adenosine was found to significantly improve cardiac function in both men and women when administered before ischemia (Shao et al., 2017). The protective effect of ischemic postconditioning (IPO) is almost identical to that of IPC. The degree of A_1_AR activation within minutes of the onset of reperfusion determined the degree of cardio protection (Solenkova et al., 2006). In studies on knockout mice, it was demonstrated that IPO-induced cardio protection can be triggered by activation of the A_1_AR on the cell membrane (Xi et al., 2008). Furthermore, A_1_AR exhibited pro-mitotic properties in coronary smooth muscle (Ahmad et al., 2009; Erices et al., 2022). A study demonstrated for the first time that activation of A_1_AR in human monocytes induced the release of vascular endothelial growth factor, thereby promoting angiogenesis (Clark et al., 2007).

#### 3.2.2 A_2_ adenosine receptor

Adenosine binds to A_2_AR on smooth muscle and endothelial cells to regulate vascular tone and has a significant vasodilator effect on both coronary and peripheral arteries. In coronary arteries, A_2_AR stimulates various signaling pathways to induce vasodilation, resulting in increased blood flow and oxygenation and producing measurable physiological parameters (Berwick et al., 2010; Guieu et al., 2015). A_2b_AR contributes more to coronary artery vasodilation in humans in disease states than in normal states. A_2_AR also plays an important role in angiogenesis. A_2_AR was observed to promote endothelial cell germination in the vascular bed of ischemic myocardium and formation of vascular networks after a certain level of adenosine during hypoxia/ischemia (Adair, 2005). In coronary artery disease (CAD), such as myocardial infarction, A_2_R may attenuate the injury caused by antiplatelet therapy. In contrast to A_2b_AR, which exerts an inhibitory effect on platelet aggregation only under stressful conditions. A_2a_AR inhibits platelet activation by activating PKA through cAMP. PKA phosphorylates specific substrates necessary for platelet activation, such as Gα_13_, which in turn inhibits RhoA/Rho kinase, ultimately inhibiting platelet activation (Johnston-Cox et al., 2011). A_2a_AR agonists down-regulate p-selectin, diminish neutrophil-platelet aggregation formation, and improve coronary no-reflow. In the porcine myocardial infarction model, the combination of ticagrelor reduced the no-reflow rate to 3.8% (He et al., 2024). Inflammation after myocardial infarction may further prolong the infarction, and the anti-inflammatory effects of A_2a_AR allow it to limit the size of the cardiac infarct at IPO. Activated A_2a_AR promotes anti-inflammatory factors and reduces generation of pro-inflammatory factors, inhibiting reactive oxygen species production by neutrophils and adhesion to endothelial cells (Carmona-Rivera et al., 2019; Zhang et al., 2024). A_2b_AR also plays a role in IPO (Li et al., 2021; Ruan et al., 2023). In addition, Dubey identified a specific role for A_2b_AR in inhibiting cardiac fibroblast proliferation and collagen synthesis, which was validated in experiments with A_2b_AR overexpression and deletion (Dubey et al., 1997). A_2b_AR participated in adenosine antiproliferation of coronary smooth muscle through the AC-cAMP-PKA axis, thereby preventing lumen narrowing and post-injury restenosis (Dubey et al., 2015; Bot et al., 2012). In summary, A_2_AR exerts a strong protective effect on the heart.

Most current investigations on the activation of A_2_AR have favored protection of the heart from injury, while studies that have blocked A_2a_AR and thus exerted a protective effect have concentrated on the nervous system, more specifically Parkinson’s disease. A_1_AR and A_3_AR negatively regulate A_2_AR-mediated coronary artery diastole, probably because A_1_AR and A_3_AR inhibit AC activity and reduce cAMP production (Talukder et al., 2002; Tawfik et al., 2006). In addition, In contrast to A_1_AR, A_2a_AR directly enhances the inotropic effects of cardiomyocytes by increasing cytoplasmic Ca^2+^ levels and myofilament Ca^2+^ sensitivity (Boknik et al., 2020). The vasodilator effects of A_2_AR may also lead to accelerate heart rate, and inhalation of adenosine in patients with asthma induces bronchoconstriction, which makes it contraindicated in patients with active asthma (Gao and Jacobson, 2017). Moreover, The A_2a_AR gene has several polymorphisms. According to studies, coronary angiography reveals a more severe degree of coronary artery stenosis, and individuals with particular A_2a_AR gene variants typically experience more severe angina pectoris symptoms (Nardin et al., 2020; Malinowski et al., 2023). In contrast to the above mentioned that in different systems or other ARs would negatively regulate the protective effect of A_2_AR, A_2b_AR itself would have a paradoxical effect. It has been shown that blocking A_2b_AR appears to be beneficial for cardiac remodeling and fibrosis. Activation of A_2b_AR also increases the metabolic activity of cardiac fibroblasts and the production of type I collagen (Bessa-Gonçalves et al., 2024). The mechanism of the profibrotic activity of A_2b_AR may be related to its mediation of proinflammatory responses. Blockade of A_2b_AR inhibited caspase-1 activity and leukocyte infiltration and attenuated the secretion of pro-fibrotic and pro-inflammatory mediators, such as transforming growth factor-beta, interleukin 6 (IL-6), and tumor necrosis factor-alpha, after myocardial infarction via the PKC-delta pathway (Alter et al., 2023; Feng et al., 2010). Compared to A_1_AR and A_3_AR, A_2_AR has a broader and more complex role, which promotes cardiac diastole, improves blood supply, and enhances cardiomyocyte tolerance to ischemia by increasing cAMP production. The complexity of A_2_AR’s action is reflected in the role of A_2b_R in cardiac remodeling, where the pro-inflammatory effect of A_2b_AR makes its role in cardiac remodeling unclear. The conflicting effects that occur with A_2b_AR itself may be due to the timing of treatment and different model systems. For instance, A_2b_AR activation lessens autophagic flux obstruction and decreases the infarct area during the early stages of reperfusion (He et al., 2024). A_2b_AR activation will encourage fibrosis and inflammation during the middle and late phases of reperfusion (Tian et al., 2023). Therefore, more research is needed to validate the optimal timing of the protective effects of A_2b_AR and to gain a deeper understanding of its indications and effector cells to avoid delaying the development of A_2b_AR due to modeling issues.

#### 3.2.3 A_3_ adenosine receptor

One of the most important topics in the realm of A_3_AR-targeted treatment is its protective role in cardiac ischemia. Numerous investigations have revealed the involvement of A_3_AR in adenosine-induced cardio protection during ischemia-reperfusion (I/R). The application of selective A_1_AR antagonists failed to terminate the protective effect of A_1_AR in IPC, leading to the discovery of A_3_AR’s role in IPC (Liu et al., 1994). However, A_3_AR is more challenging to research because of its low expression level, making it difficult to determine its function in the heart. The expression of A_3_AR in cardiomyocytes was verified using various agonists and antagonists. During reperfusion, A_3_AR reduced additional harm to heart tissue by having anti-inflammatory properties (Ge et al., 2010). Activation of A_3_AR reduced cardiac infarct size in I/R mice and mediated pro-survival signaling pathways, such as phosphoinositide 3-kinase/PKB pathways and extracellular signal-regulated kinase 1/2 (Hussain et al., 2014). A_3_AR also protected against cardiovascular damage by promoting myocardial ATP-sensitive potassium channel opening to protect against myocardial I/R injury, and this protective action was absent in A_3_AR knockout mice (Wan et al., 2019). The receptor has to be well connected to protective intracellular signaling pathways because of its potent cardioprotective effect and low cardiac expression of A3AR. Additionally, since immune cells express A_3_AR at high levels and cardiomyocytes express it at relatively low levels, indirect protection may be possible (Garcia-Garcia et al., 2021; Pasquini et al., 2021). In terms of vasodilation, genetic deletion of A_3_AR or antagonism of A_3_AR increases coronary blood flow (Talukder et al., 2002). A_3_AR can lower blood pressure and heart rate, but under normal adenosine concentrations, the role of A_3_AR in regulating blood pressure changes was suppressed by the vasodilatory signal of A_2a_AR (Zhao et al., 2000).

Maintaining ideal heart function requires balancing A_3_AR expression levels. A_3_AR overexpression leads to reduced heart rate, energy conservation, and protection from ischemic injury; however, elevated expression of A_3_AR connected to dilated cardiomyopathy development (Black et al., 2002). A_3_AR-mediated cardio protection remains controversial. Studies in mice with disrupted A_3_AR genes showed smaller myocardial infarct size and improved cardiac function (Guo et al., 2001). A_3_AR’s paradox could be caused by species-specific variations. Through mast cell degranulation, A_3_AR signaling may cause a pro-inflammatory response in mast cells, particularly in rodents, which can harm the heart (Garcia-Garcia et al., 2021; Pasquini et al., 2021; Koda et al., 2010). Thus, mice deficient in A_3_AR might benefit heart function. These findings highlight how intricate A_3_AR signaling is. The timing of A_3_AR-mediated cardio protection is also controversial, and the debate continues as to whether pre- or post-ischemic use of A_3_AR agonists is superior (Borea et al., 2015; Nisha et al., 2016). A_3_AR’s protective effect on the myocardium may be affected by genetic polymorphisms that disrupt its normal function, such as enlarging the infarction region in myocardial infarction patients (He et al., 2018). In addition, it was proposed that the blood pressure- and heartrate-lowering effects of A_3_AR are suppressed by A_2a_AR at normal adenosine concentrations, but in pathological conditions, there are conflicting effects between the two receptors. So far, only a few of A3AR agonists have entered clinical trials for the treatment of heart disease, highlighting the gap between preclinical and clinical applications of A3AR agonists in cardiovascular medicine.

## 4 Adenosine receptors as diagnostic and therapeutic targets in cardiovascular system

Cardiovascular illnesses can be diagnosed and treated by targeting adenosine receptors (ARs), which have important physiological and pathological roles in the cardiovascular system. From a diagnostic standpoint, determining the degree of AR expression or activity in cardiovascular tissues aids in determining the severity and course of cardiovascular disorders. In terms of treatment, certain progress has been made in the drug research and development for ARs. Selective ARs agonists or antagonists can regulate cardiovascular function and improve disease conditions (Table 2).

**TABLE 2:** Therapeutic roles of ARs in CVDs.

Heart diseases	Models	Drug	Target	Category	Objective	Mechanism	Outcome	Ref.
AF	Atrial myocytes	CGS21680	A_3_AR	Agonist	Therapeutic	Counteracts excessive A2AR activation	Inhibition of spontaneous calcium release thereby reducing the risk of AF development	Tarifa et al. (2023)
AF	Rat hearts	PSB36	A_1_AR	Antagonist	Therapeutic	Prolonged AP duration at 90% of repolarization and effective refractory period in rat atria	Prevented AF events and reduced AF duration	Soattin et al. (2020)
AF	Patients with AF	Adenosine	—	Agonist	Diagnostic	Intravenous adenosine restores conduction in viable but temporarily nonconducting pulmonary veins	Adenosine test identifies occult conduction; targeted ablation helps improve success rate	Macle et al. (2015)
HF	Chronic HF dogs	DPCPX	A_1_AR	Antagonist	Therapeutic	—	Elimination or prevention of adenosine-induced sinoatrial node dysfunction and AF	Lou et al. (2014)
HF	Patients with congestive HF	Naxifylline	A_1_AR	Antagonist	Therapeutic	Decreases renal blood flow and glomerular filtration rate	Increases urinary sodium and diuresis	Gottlieb et al. (2002)
HF	Patients with AHF	Rolofylline	A_1_AR	Antagonist	Therapeutic	Decreases renal blood flow and glomerular filtration rate	Increased urine output and improved renal function	Voors et al. (2011)
HF	Patients with HFpEF	Neladenoson bialanate	A_1_AR	Agonist	Therapeutic	Improvement of mitochondrial function and enhancement of SERCA2a activity	Improving cardiac structure and function in heart failure patients	Shah et al. (2019)
AS	ApoE−/−mice	DPCPX	A_1_AR	Antagonist	Therapeutic	Reduced IL-5, IL-6, and IL-13 concentrations	Reduces atherosclerotic lesions	Teng et al. (2014)
AS	ApoE−/−mice	Istradefylline	A_2a_AR	Antagonist	Therapeutic	Inhibition of endothelial-to-mesenchymal transition	suppresses atherosclerosis *in vivo*	Cai et al. (2024)
AS	ApoE−/−mice	BAY 60-6,853	A_2b_AR	Agonist	Therapeutic	Reduces sterol regulatory element binding protein-1 and its 2 downstream targets levels	Reduces lipid levels and AS	Koupenova et al. (2012)
CAD	Healthy subjects	Adenosine	A_2_R	Agonist	Diagnostic	Increases cAMP production and relaxes smooth muscle cells	Increased perfusion contrast between normal and stenotic areas	Lassen et al. (2022)
CAD	Patients with MPI	Dipyridamole	PDE	Inhibitor	Diagnostic	Inhibits PDE activity and increases adenosine levels	Diagnosing myocardial ischemia and coronary artery disease	Giorgi et al. (2017)
CAD	Patients with regadenoson stress	Regadenoson	A_2a_AR	Agonist	Diagnostic	Increased cAMP levels, phosphorylate PKA and hyperpolarize membranes	Dilates coronary arteries and increases coronary blood flow	Khan et al. (2022)
MI	SHR-MI rats	LASSBio-294	A_2a_AR	Agonist	Therapeutic	Reduction of collagen deposition and tumor necrosis factor α expression in the left ventricle	Prevented the progression of cardiac dysfunction	da et al. (2017)
MI	MIRI rats	BAY-60-6,583	A_2b_AR	Agonist	Therapeutic	Mitigating impaired autophagic flux and excessive endoplasmic reticulum stress	Alleviates MIRI	He et al. (2024)
Hypertension	Hypertensive mice	CGS21680	A_2a_AR	Agonist	Therapeutic	Increased lymphatic capillary density and activation of Mitogen- and stress-activated kinase 1	Mediation of lymphangiogenesis to prevent salt-sensitive hypertension	Zhuang et al. (2023)
Hypertension	WT mice	DPCPX	A_1_AR	Antagonist	Therapeutic	Reduced vasoconstrictor responses to angiotensin II	May result in lower blood pressure	Yadav et al. (2015)
I/R	Isolated, buffer-perfused heart	CCPA	A_1_AR	Agonist	Therapeutic	Phosphorylation of myocardial epidermal growth factor receptor, and PKB	Improved recovery from ischemia and a 50% reduction in left ventricular diastolic dysfunction	Williams-Pritchard et al. (2011)
I/R	Isolated, buffer-perfused heart	CP-532,903	A_3_AR	Agonist	Therapeutic	Activation of ATP-sensitive potassium channel	Increases ischemic tolerance and protects the heart	Wan et al. (2019)
I/R	I/R dog	IB-MECA	A_3_AR	Agonist	Therapeutic	—	Both pre-coronary occlusion and pre-reperfusion administration reduce myocardial infarct size	Auchampach et al. (2003)
		CCPA	A_1_AR	Agonist	Therapeutic	—	Reduces heart rate and systemic blood pressure and increases coronary blood flow	

### 4.1 Atrial fibrillation

Atrial fibrillation (AF) is the most common arrhythmia, involving 1%–4% of the population, and prolonged AF can cause a series of adverse clinical outcomes such as stroke and heart failure (HF), with stroke being the most important cause of death and disability in AF (Andrade et al., 2020). ARs are therapeutic and diagnostic targets for AF.

In high-risk patients, AF can be induced by adenosine (Ip et al., 2013). The right atria of high-risk patients have shown significant expression of G-protein-coupled inwardly rectifying potassium channels and heterogeneous expression of A1R, indicating that localized reentry in the right atria is the cause of adenosine-induced AF (Li et al., 2016). In a canine model, adenosine-induced anomalies in atrioventricular node conduction and shortening of atrial repolarization were linked to elevated A1AR expression in the atrioventricular node and atrial cardiomyocytes, which raised the risk of atrial fibrillation (Lou et al., 2014). The isolated hearts of rats treated with [1-butyl-3-(3-hydroxypropyl)-8-(3-noradamantyl)xanthine (PSB36) and [2-chloro-*N* ^6^-cyclopentyladenosine (CCPA) shortened and prolonged action potential duration at 90% of repolarization and effective refractory period, respectively, suggesting that antagonizing A_1_AR with PSB36 prevented AF event and shortened AF duration (Soattin et al., 2020). A_2a_AR and A_2b_AR expression was significantly upregulated in the left atrium after AF surgery (Maille et al., 2021). A_2a_AR was pro-arrhythmic by promoting spontaneous calcium release, whereas activation of A_3_AR reduced A_2a_AR-mediated spontaneous calcium release in human atrial myocytes (Tarifa et al., 2023). In addition, A_2b_AR may be associated with the regulation of fibrosis in AF (Maille et al., 2022). The two-sided regulatory role of ARs is well illustrated by the fact that different subtypes of ARs exert or promote or inhibit the development of AF. In addition, the adenosinergic system has a diagnostic value for AF.

Pulmonary vein ectopic pulsation has been identified as a key AF trigger and maintenance mechanism, laying the theoretical foundation for pulmonary vein ablation for AF, but the rate of postoperative recurrence remains high (Haïssaguerre et al., 1998). Most of the recurrent cases were attributed to the recovery of concealed conduction between the pulmonary vein and the left atrium, which led to failure of pulmonary vein isolation. Following pulmonary vein isolation, the adenosine test provides a strong predictive value for AF recurrence. Arentz et al. were the first to report a possible association between adenosine-induced occult conduction in the pulmonary veins and AF recurrence (Arentz et al., 2004). In recent years, the adenosine test has been used clinically to screen for the restoration of pulmonary vein conduction, with the aim of guiding ablation strategies and improving success rates. Large and small-scale studies have shown that the adenosine testing can improve the success rate of ablation, but there are still many limitations in the use of the adenosine test in the post-ablation period: discrepancies between the evaluation of ablation styles and indices in different centers, the adenosine dosage and the time of administration have not yet been standardized as well as the drug’s adverse effects and the time-consuming operation, etc.,. (McLellan et al., 2017; Macle et al., 2015). Further high-quality studies are needed to clarify the specific clinical use and possible benefits of the adenosine tests.

### 4.2 Atherosclerosis

Atherosclerosis (AS) is a progressive, long-term condition of large and medium-sized arteries marked by the formation of atherosclerotic plaques and is a major cause of cardiovascular disease (Libby, 2021). With the formation, enlargement, and accumulation of foam cells, hypoxia and inflammation are exacerbated, adenosine levels are elevated, and they are involved in AS. Based on the multiple effects of ARs, the treatment of AS requires inhibition of AS progression by antagonizing or activating different subtypes.

When DPCPX, an A1AR antagonist, was administered to apolipoprotein E knockout (ApoE^−/−^) mice, atherosclerotic lesions decreased along with the levels of plasma IL-5, IL-6, and IL-13. This could be connected to A1AR’s pro-inflammatory and pro-mitotic characteristics. (Teng et al., 2014). A_2a_AR inactivation similarly protect ApoE−/−mice from AS. AS was suppressed and macrophage numbers were reduced in double knockout mice produced by crossing ApoE−/−mice with A_2a_AR knockout mice compared to ApoE−/−mice (Wang et al., 2009). This is due to macrophage and foam cell death resulting from increased p38 mitogen-activated protein kinase activity in A_2a_AR knockout mice. Endothelium-specific A_2a_AR deficiency or blockade of A_2a_AR with Istradefylline inhibited/attenuated AS in ApoE−/−mice (Cai et al., 2024). In contrast to antagonizing the protective role of A_1_AR and A_2a_AR in AS, A_2b_AR deficiency led to more pronounced AS in ApoE−/−mice, and *in vivo* administration of the A_2b_AR agonist BAY 60-6,853 reduced lipid levels to inhibit AS (Koupenova et al., 2012).

### 4.3 Coronary artery disease

The hardening of coronary blood arteries is a hallmark of CAD, thereby leading to myocardial ischemia, hypoxia, or necrosis. Since CAD is the world’s leading cause of death and disability, early detection and treatment are crucial (Ralapanawa and Sivakanesan, 2021).

In CAD, an imbalance between oxygen supply and demand leads to adenosine accumulation during ischemia. The regulatory role of the adenosinergic system in CAD was first considered and investigated at the beginning of the 21st century, and the development of highly potent and selective A_2a_AR agonists has been the subject of chemical research in the ensuing 30 years (Klotz, 2000; Cristalli et al., 2003; Paganelli et al., 2021). Adenosine, as a sensitive marker of myocardial ischemia, is a useful tool for CAD patients during myocardial imaging (Lassen et al., 2022). Adenosine-loaded myocardial perfusion imaging is based on imaging abnormalities in myocardial cell perfusion or metabolism. Intravenous adenosine binding to the A_2_AR rapidly dilates coronary arteries and increases myocardial blood flow reserve, whereas failure of diseased coronary arteries to dilate accordingly results in increased perfusion contrast between normal and stenotic coronary regions of the heart (Paganelli et al., 2021; Garcia-Dorado et al., 2014). In pharmacologic stress tests, dipyridamole, a vasodilator, increased coronary vasodilation and coronary blood flow via A_2a_AR while inhibiting adenosine uptake and metabolism (Gaudry et al., 2020). Regadenoson, a selective A_2a_AR agonist, was approved by the U.S. Food and Drug Administration in 2008 for use in myocardial perfusion imaging (MPI) and exercise contraindications. It is more frequently employed in clinical practice because of its quick start of action, short duration that is enough to produce a congestive response, efficacy that is comparable to that of adenosine, and lack of negative reactions and side effects (Al Jaroudi and Iskandrian, 2009).

ARs are mainly used to treat CAD through antiplatelet activation. A_2a_AR expressed on platelets plays a significant role in the inhibition of platelet aggregation (Wolska and Rozalski, 2019). 5′-N-ethylcarboxamidoadenosine, CGS21680, and ATL-146e both exerted antiplatelet aggregation effects by binding to A_2a_AR (Clark et al., 2019; Linden et al., 2008; Fuentes et al., 2014). By altering the activation of the AC-cAMP-PKA pathway, 1.8-cineole (A_2a_AR agonist) prevented the expression, release, and platelet aggregation of platelet activation indicators (Petry et al., 2024). Dipyridamole is anti-platelet aggregation by inhibiting platelet PDE, leading to the accumulation of cAMP and guanine cyclic phosphate (Kim and Liao, 2008). However, due to the lack of sufficient evidence to support its use for various indications and with the emergence of newer antiplatelet agents, the use of dipyridamole in CAD has been out of practice (Ye et al., 2010). It is clear from the above that lower A_2a_AR levels favor platelet aggregation in acute coronary syndromes. Conversely, drugs that increase plasma adenosine levels can inhibit platelets. According to the results of an investigation, AR agonists (regadenoson, NECA, LUF5835) and P2Y_12_ antagonists (cangreor or prasugrel metabolite) work together to limit platelet activity more effectively than P2Y_12_ antagonists alone (Wolska et al., 2019). Moreover, ARs agonists have a more important role in people who react badly to P2Y_12_ inhibitors. This finding offers a useful foundation for the clinical management of platelet aggregation with combination medications.

ARs may also play a cardioprotective role in ischemia and reperfusion through IPC and IPO, and a large number of reports on the cardioprotective aspects of AR-mediated cardio protection have appeared since the 1990s. Although the research results on whether administering adenosine in the coronary artery before direct PCI can reduce the size of myocardial infarction are inconsistent, adenosine treatment for patients with shorter ischemic times undergoing direct percutaneous coronary intervention can salvage more myocardium and is advantageous for improving left ventricular ejection fraction (Polimeni et al., 2016). All four ARs can reduced infarct size and protected the heart through IPC and IPO (Zhang et al., 2024; Ruan et al., 2023; Wan et al., 2019; Williams-Pritchard et al., 2011). However, when cardioprotective therapies in humans, a number of comorbidities, including obesity, diabetes, and hypercholesterolemia, are prevalent in comparison to healthy laboratory animals. Given this circumstance, additional information is required to design medicine (Lasley, 2018).

### 4.4 Heart failure

HF is a clinical syndrome that results in elevated intracardiac pressure and decreased cardiac output both under stress and at rest. Using classic diuretics to treat HF usually induces or exacerbates kidney damage. Additionally, heart failure with preserved ejection fraction (HFpEF) also lacks effective treatment (McMurray et al., 2012; Ponikowski et al., 2016). Drugs that antagonize A_1_AR to exert a diuretic effect and activate A_1_AR to provide cardio protection have been developed to address these problems.

A_1_AR antagonists, such as naxifylline and rolofylline, have been used in clinical phase II and III trials. Antagonism of A_1_AR improves glomerular filtration by dilating afferent small arterioles and blocking interglomerular feedback. Clinical trial studies of naxifylline and rolofylline have demonstrated that combining them with diuretics reduces the collateral diuretic dosage (Gottlieb et al., 2002). In a large phase III study, rolofylline had a modest diuretic effect in patients with acute HF (AHF) with mild to moderate renal insufficiency but did not prevent sustained deterioration of renal function in patients with AHF with volume overload and renal insufficiency and had a higher incidence of seizures and stroke (Voors et al., 2011). Further evaluation of its efficacy and safety in phase IV clinical trials is needed to provide more options for diuretic therapy in HF patients. Neladenoson bialanate, a drug targeting A_1_AR for the treatment of HFpEF, is currently in clinical phase II trials. Without the negative side effects of full A1AR agonists or A1AR antagonists, Neladenoson bialanate, a partial adenosine A1AR agonist, has been demonstrated in preclinical models to better mitochondrial function, perform energy substrate utilization, increase serca2a activity, reverse ventricular remodeling, and supply anti-ischemic cardio protection. No significant dose-response relationship was detected for nelladenoson bialanate in HFpEF patients in terms of changes in exercise capacity from baseline to 20 weeks. In light of these findings, if Neladenoson bialanate is further developed for the treatment of patients with HFpEF, new approaches will be required (Shah et al., 2019).

### 4.5 Hypertension

Persistent systemic arterial hypertension is the hallmark of hypertension and is the most important modifiable factor in cardiovascular morbidity and mortality worldwide (GBD 2019 Risk Factors Collaborators, 2020). The pathophysiology of hypertension involves ARs, and various AR subtypes have distinct effects on blood pressure regulation.

A_2a_AR is abundantly expressed in multiple cell types and regulates cardiovascular responses. A_2a_AR activation reduced hypertension by promoting vasodilation, controlling adipokine secretion, preventing immune cells from releasing inflammatory substances, and promoting cardiovascular homeostasis (Quast et al., 2017; Ruan et al., 2018). In Dahl salt-sensitive hypertensive animals, deletion of A_2a_AR led to salt-induced elevation of blood pressure (Jackson et al., 1979). Pre-eclampsia also led to elevated blood pressure in mothers. High levels of adenosine have been found in the fetal-placental circulation in preeclamptic pregnancies, which may be related to A_2a_AR-NO signaling (Escudero et al., 2013). According to recent research, a pathogenic etiology of hypertension may be sodium and fluid imbalance brought on by defective lymphangiogenesis and lymphatic dysfunction. Activation of A_2a_AR using CGS21680 reduced blood pressure by increasing lymphatic capillary density in mice (Zhuang et al., 2023). Activation of A_1_AR in proximal tubules led to excess Na^+^ reabsorption, resulting in Na^+^ retention, which induced hypertension (Lee et al., 2012). In one study, adenosine alone versus a combination of adenosine and caffeine resulted in no change in renin secretion, presumably due to the counteracting effects of both A_1_AR and A_2a_AR, but the inference needs to be confirmed by further studies (Wierema et al., 2005). In the aorta of hypertensive mice with high A_1_AR levels, the A_1_AR agonist CCPA induced more pronounced vasoconstriction (Yadav et al., 2019). In addition, the protective effects of A_1_AR and A_2b_AR against hypertension were different in different sexes of hypertensive model rats. A recent experiment showed that deletion of A_1_AR and A_2b_AR was protective against salt-induced hypertension in female rats and that this protective effect was overcome when the salt diet reached extremely high levels (Jackson et al., 1979). Perhaps when the salt diet reaches a certain level, the renal ARs in pathophysiology as well as their excretion function as well as the degree of inflammatory activation may counteract the antihypertensive effect of the A_1_AR deletion, leading to treatment failure. The different effects of ARs in therapy described above demonstrate the complexity of ARs and suggest that future therapeutic strategies need to take into account the specificity of AR subtypes as well as their changing roles in different pathologic states.

## 5 Discussion and perspective

This paper systematically summarizes the complex regulatory roles of adenosine and its receptors (ARs) in the cardiovascular system, highlighting their potential value in the prevention and treatment of cardiovascular diseases. Adenosine exerts significant physiological and pathological effects through its four receptor subtypes. Studies have shown that the roles of different AR subtypes in the cardiovascular system can be both synergistic and antagonistic. Furthermore, even the same receptor subtype may exhibit paradoxical effects under different pathological conditions, such as the protective role of A_1_AR in ischemic preconditioning versus its pro-fibrotic effects, or the dual regulatory functions of A_2b_AR in cardiac remodeling. These “paradoxes” not only reflect the complexity of AR signaling but also point to new opportunities for precision medicine in cardiovascular diseases.

Despite the substantial potential of ARs in the diagnosis and treatment of CVDs—including myocardial ischemia, heart failure, hypertension, atrial fibrillation, and atherosclerosis—their clinical translation faces several challenges. Although adenosine and its receptor agonists have been shown in several animal studies to reduce IRI, these preclinical effects have not been consistently replicated in human clinical trials. Adenosine administered intravenously decreased the area of myocardial infarction in the Acute Myocardial Infarction Study of Adenosine II trial, but it had no effect on clinically significant endpoints, including death, reinfarction, or the rate of hospitalization for heart failure. One reason for this is that the receptor desensitization process in chronic human disorders cannot be replicated in clinical models. However, the protective effect of adenosine may be diminished if the PI3K/Akt pathway is weakened due to mitochondrial function impairment in aged or diabetic patients. Nevertheless, such intricate disease underpinnings are absent from conventional animal models. There are variations in the distribution. The distribution and affinity of receptors also vary by species, with the human A_3_R having a much higher affinity for adenosine than rats, the animal model requiring a higher dose to activate the protective pathway, and the polymorphism of rs35511654 in the human A_3_AR gene reducing the receptor sensitivity in 30% of the population. Unfortunately, preclinical models cannot replicate this heterogeneity, which leads to bias in drug response prediction. The development of agonists that can specifically target a particular subtype of AR is extremely difficult due to the structural and functional similarities among these subtypes. Effective binding with the target receptor subtype and avoiding cross-reactions with other subtypes must be taken into account when building pharmacological molecules; failing to do so could result in a rise in adverse reactions or complicated and unpredictable therapeutic effects. Consider creating a drug delivery system that can target and deliver nanoparticles to sick tissues in order to lessen a number of systemic side effects brought on by conventional drug delivery techniques. The evaluation of targeting efficiency, specificity, and safety are among the difficulties this measure faces.

Studying the variability of AR expression in cardiovascular tissues using single-cell sequencing and spatial transcriptomics can reveal new information about receptor dynamics and disease mechanisms. Adenosine receptor expression patterns and levels in various cell types (e.g., cardiomyocytes, endothelial cells, macrophages, neutrophils, etc.) in cardiovascular tissues can be determined by single-cell sequencing. Additionally, new cell subsets and their unique gene expression characteristics can be identified, and the changes in adenosine receptor expression in various cell types over the course of cardiovascular diseases can be dynamically monitored. Adenosine receptor functions and regulatory mechanisms can be studied at the tissue level thanks to spatial transcriptomics, which can accurately identify the expression positions of adenosine receptors, reveal their distribution characteristics in cardiovascular tissues, analyze the spatial relationships and interactions among various cell types, and create high-resolution expression maps of adenosine receptors in cardiovascular tissues. In order to fully investigate the intricate mechanism of action of adenosine receptors in cardiovascular disorders, future studies can further combine multi-omics approaches.

Overall, by delving into the complexity of different AR subtypes and their interactions, we can better understand how to utilize these molecular mechanisms to enhance therapeutic effects while avoiding potential side effects. Therefore, ongoing research on ARs is expected to provide scientific evidence and new perspectives for innovative drug development and personalized treatment, driving the treatment of CVDs towards precision and individualization.
